# Supplementary material for: Curcumin-Loaded Platelet Membrane Bioinspired Chitosan-Modified Liposome for Effective Cancer Therapy
Source: Pharmaceutics. 2023 Feb 13;15(2):631. doi: 10.3390/pharmaceutics15020631 (PMC9965064; doi:10.3390/pharmaceutics15020631)
Supplement: Supplementary file 1 [file pharmaceutics-15-00631-s001.zip › pharmaceutics-2131093-supplementary.pdf]

Supplementary materials

Curcumin-loaded Platelet Membrane Bioinspired Chitosan-modified Liposome for Effective Cancer Therapy

Shengli Wan [1,2,3,4,†], Qingze Fan [2,4,†], Yuesong Wu [4,5], Jingqing Zhang [6], Gan Qiao [4,5], Nan Jiang [1,4], Jie Yang [1,4], Yuanzhi Liu [1,2,4], Jingyan Li [1,4], Sawitree Chiampanichayakul [1], Singkome Tima [1,7], Fei Tong [4,5], Songyot Anuchapreeda [1,7,*], and Jianming Wu [3,5,*]

1 Division of Clinical Microscopy, Department of Medical Technology, Faculty of Associated Medical Sciences, Chiang Mai University, Chiang Mai 50200, Thailand

2 Department of Pharmacy, The Affiliated Hospital of Southwest Medical University, Luzhou 646000, China

3 School of Basic Medical Sciences, Southwest Medical University, Luzhou 646000, China

4 School of Pharmacy, Southwest Medical University, Luzhou 646000, China

5 Key Laboratory of Medical Electrophysiology of Ministry of Education of China, School of Pharmacy, Southwest Medical University, Luzhou 646000, China

6 Chongqing Research Center for Pharmaceutical Engineering, Chongqing Medical University, Chongqing 400016, China

7 Center for Research and Development of Natural Products for Health, Chiang Mai University, Chiang Mai 50200, Thailand

* Correspondence: songyot.anuch@cmu.ac.th (S.A.); jianmingwu@swmu.edu.cn (J.W.)

†These authors made equal contributions to this work.

SUPPORTING FIGURES

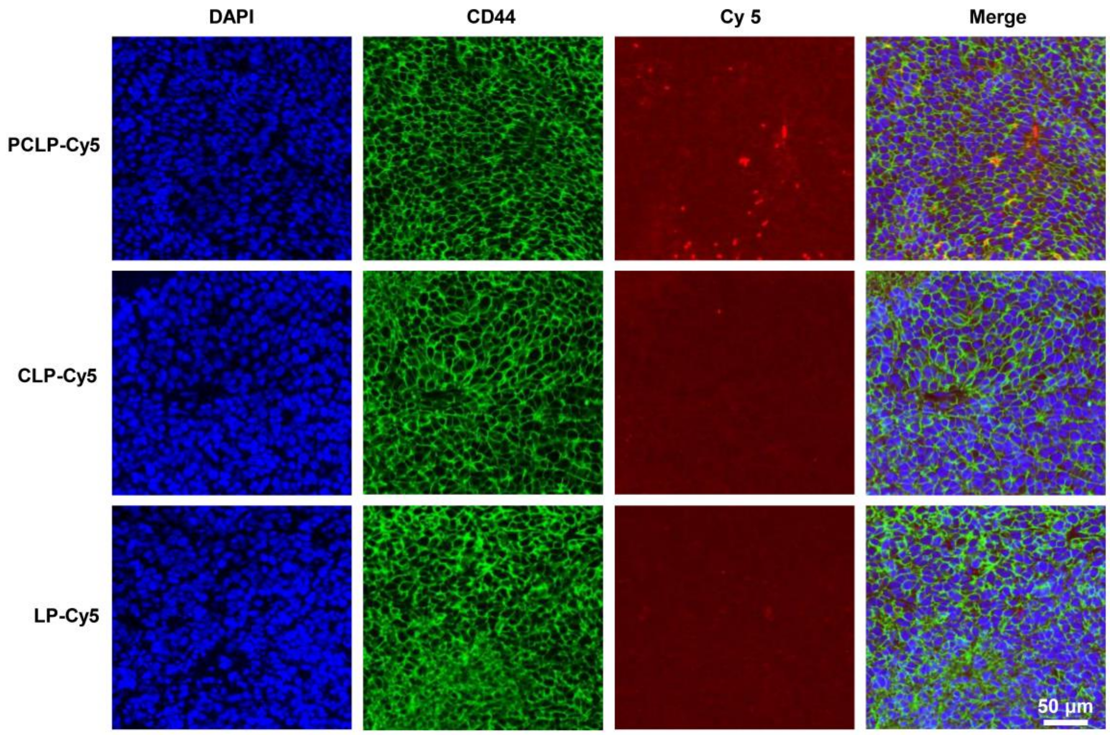

Figure S1. Localization of vesicles in tumor tissues. HepG2 tumor-bearing mice were intravenously injected with PCLP-Cy5, CLP-Cy5 or LP-Cy5. After treatment for 24 h, the tumors were collected and sectioned, followed by staining using an antibody against CD44 (green), and the nuclei were labeled using DAPI (blue). The nanoparticles were labeled with Cy5 (red).

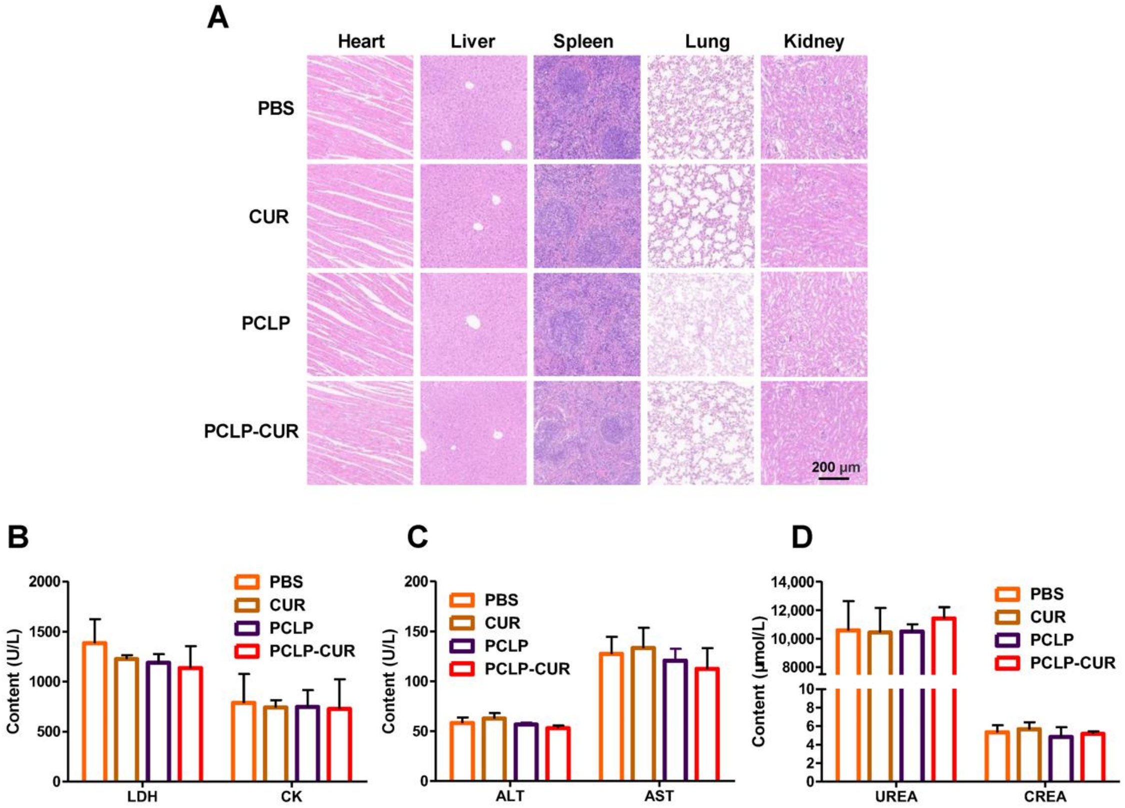

Figure S2. In vivo safety of various formulations in healthy BALB/c nude mice. (A) H & E-stained slices of the major organs after treatment. Biochemical analysis of (B) LDH and CK, (C) ALT and AST, and (D) UREA and CREA in mice treated with different formulations (n = 3). ALT: alanine aminotransferase; AST: aspartate aminotransferase; CK: creatine kinase; CREA: creatinine; LDH: lactate dehydrogenase; UREA: urea.
